# AI‐Enabled Automated Schistocyte Classification in Peripheral Blood for TMA Auxiliary Diagnosis

**DOI:** 10.1111/ijlh.70209

**Published:** 2026-08-05

**Authors:** Lei Shang, Chao Fang, Dongshuo Li, Xuesong Wang, Jiani Yu, Yunchu Cui, Jingliang Chen, Xuekai Liu

**Affiliations:** ^1^ Department of Clinical Laboratory Aerospace Center Hospital Beijing China; ^2^ Faculty of Engineering and Design University of Auckland Auckland New Zealand; ^3^ School of Science, Beijing Jiaotong University Beijing China; ^4^ School of Public Health, Xiamen University Xiamen China

**Keywords:** artificial intelligence, deep learning, image classification, image segmentation, peripheral blood smear, ResNet, schistocytes, thrombotic microangiopathy (TMA), Xception

## Abstract

**Background:**

Schistocytes are critical morphological markers for thrombotic microangiopathy (TMA) diagnosis. Manual identification is hampered by inconsistent standards and high interobserver variability, compromising accuracy. Although AI has advanced in hematology, AI‐enabled schistocyte classification remains under‐studied, creating a clinical gap.

**Methods:**

We constructed a two‐stage AI system for segmentation and classification. Three segmentation models (U‐Net, ENet, and R2U‐Net) were assessed on 25 067 RBCs from 183 patients. ResNet‐50 and Xception were trained on 28 586 RBCs, including 13 125 ICSH‐classified schistocytes, using grayscale and RGB images. Clinical validation was performed on 156 784 RBCs from 219 patients. Performance was evaluated using recall, specificity, precision, and *F*_1_ score.

**Results:**

R2U‐Net achieved the best segmentation (recall = 0.868, *F*_1_ = 0.881, mIoU = 0.807). The Xception‐RGB model performed best in classification (weighted *F*_1_ = 0.957), with high precision and recall for schistocyte subtypes. Clinical validation showed excellent reliability with all weighted metrics at 0.998.

**Conclusions:**

This two‐stage framework enables accurate schistocyte analysis with better feature representation using RGB images and Xception. It improves diagnostic accuracy and reproducibility, supporting TMA auxiliary diagnosis and clinical decision‐making.

## 1 Introduction

Schistocytes are cell fragments generated by mechanical destruction of red blood cells (RBCs) in the systemic circulation. Their detection provides crucial morphological clues for diagnosing TMA, serves as a prognostic indicator for TMA following hematopoietic stem cell transplantation, and is associated with conditions including artificial heart valves, HELLP syndrome, malignant hypertension, and tumor metastasis [1, 2, 3].

Schistocyte testing mainly includes automated hematology analyzer screening and manual microscopic examination [4]. Automated FRC measurement screens tiny erythrocyte fragments smaller than 30 fL via cell volume and light scatter parameters without morphological discrimination, which commonly yields higher counts than manual microscopy. Per ICSH recommendations, FRC serves only as an initial screening indicator for TMA, and all positive FRC results require confirmatory peripheral blood smear review. Manual morphological identification includes only angular cells, helmet cells, small fragmented RBCs, and microspherocytes as schistocytes, while genuine bite cells, acanthocytes, and other poikilocytes are excluded. Morphology, marginal contour, and central pallor constitute the core diagnostic criteria [5].

Microscopic examination of routinely stained peripheral blood smears remains the clinical gold standard for schistocyte identification. Although unified morphological criteria issued by ICSH [6, 7] and JSLH [8] are available, inconsistent training levels among laboratory staff, variable guideline compliance, and uneven smear quality lead to prominent interlaboratory reporting discrepancies, adversely affecting clinical diagnosis and patient outcomes. In addition, differences in counting protocols, screening fields, counted RBC ranges, and auxiliary instruments further induce interobserver counting bias [9].

Per ICSH morphological guidelines, schistocytes were categorized into four subtypes in this study: (1) Angular cells (damaged RBCs with sharp‐angled projections > 3 μm in diameter); (2) Helmet cells (RBCs with a relatively smooth cut edge on one lateral margin post‐damage); (3) small fragmented RBCs (sharp‐angled fragments < 3 μm in diameter); and (4) microspherocytes (pallor‐lacking cells representing secondary fragmentation changes, included only when the other three subtypes are concurrent) (Figure S1).

In recent years, AI‐assisted hematological cell morphology analysis has advanced rapidly, with deep learning showing particular promise in hematopoietic cell recognition [10, 11]. While numerous studies have focused on AI‐based leukocyte identification, few have addressed RBC morphology analysis—especially schistocyte classification [12]. To address this research gap, we developed a deep learning‐based intelligent model for efficient and accurate schistocyte classification and enumeration in large‐scale peripheral blood smear images. Strictly following the authoritative ICSH morphological guidelines for schistocyte identification, this model aims to improve the accuracy and reproducibility of schistocyte detection and promote the clinical implementation of standardized ICSH‐compliant schistocyte classification.

## 2 Materials and Methods

### 2.1 Ethics Approval and Patient Consent

This study was approved by the Ethics Committee of Aerospace Center Hospital (Approval no. 2023(122), Date: 2023‐10‐08) and conducted in accordance with the Declaration of Helsinki. Written informed consent was obtained from all enrolled individuals.

### 2.2 Patient Enrollment and Sample Selection

#### 2.2.1 Segmentation Model Dataset

A total of 183 peripheral blood smears were collected from 183 individuals undergoing routine health check‐ups. Smears were digitized by scanning and subjected to strict quality control (QC) screening (excluding contaminated smears, those with severe cell overlap, or uneven staining). An RBC segmentation dataset containing 25 067 cells was established and partitioned into a training set (19 368 RBCs, 77%) and test set (5699 RBCs, 23%) (Table S1).

#### 2.2.2 Classification Model Dataset

A total of 10 peripheral blood smears were collected from 3 patients diagnosed with TMA. The inclusion criterion was a schistocyte proportion > 10% per smear to ensure high‐quality atlas library construction. After strict screening, a dedicated dataset of 28 586 RBCs was established, including 13 125 schistocytes (distributed across 4 subtypes) and 15 461 normal RBCs. The dataset was split into a training set (24 278 cells, 85%) and a test set (4308 cells, 15%) (Table S2).

#### 2.2.3 Clinical Validation Dataset

A total of 219 peripheral blood smears were enrolled from 219 clinical patients. All samples were flagged as “contains schistocytes” by the Mindray BC‐6800 Plus automated hematology analyzer during routine complete blood count (CBC) testing. After strict QC screening, a clinical validation dataset of 156 784 RBCs was established, including 3846 schistocytes (4 subtypes) and 152 938 normal RBCs (Table S3).

### 2.3 Blood Smear Preparation

Peripheral blood specimens (2 mL EDTA‐K2 anticoagulated venous whole blood) were collected under fasting conditions and thoroughly mixed immediately post‐sampling. Smears were prepared using the Mindray SC‐120 smear‐making and staining analyzer, with parameters (blood droplet volume: 2–4 μL; smear angle: 20.0°–36.0°; speed: 30–185 mm/s; spreading time: 0.5–3.0 s) adjusted based on hematocrit or viscosity. Staining included methanol prefixation, primary staining with undiluted solution, counterstaining with buffered staining solution, rinsing, and drying.

### 2.4 Digital Workflow for Blood Smears

Smears were numbered and scanned using the DCS‐1600 Cellular Medical Image Analyzer (Inlabo [Beijing] Medical Technology Service Co. Ltd.) at 2048 × 2448 pixel resolution. Total scanning and analysis time per smear was 10 min. Although the software allows manual selection of scanning position and area, scanning parameters were fixed in this study. Image acquisition was uniformly performed at the region one‐third inward from the feather edge of each peripheral blood smear, with a constant single‐frame capture size of 3 mm × 3 mm (Figure S2).

### 2.5 Standards for Segmentation and Classification of Schistocytes

RBC contour delineation was performed using the DCS‐1600 Cellular Medical Image Analyzer, with manual corrections applied to regions with inaccurate cell identification or imprecise cell contours (Figure 1).

**FIGURE 1:**
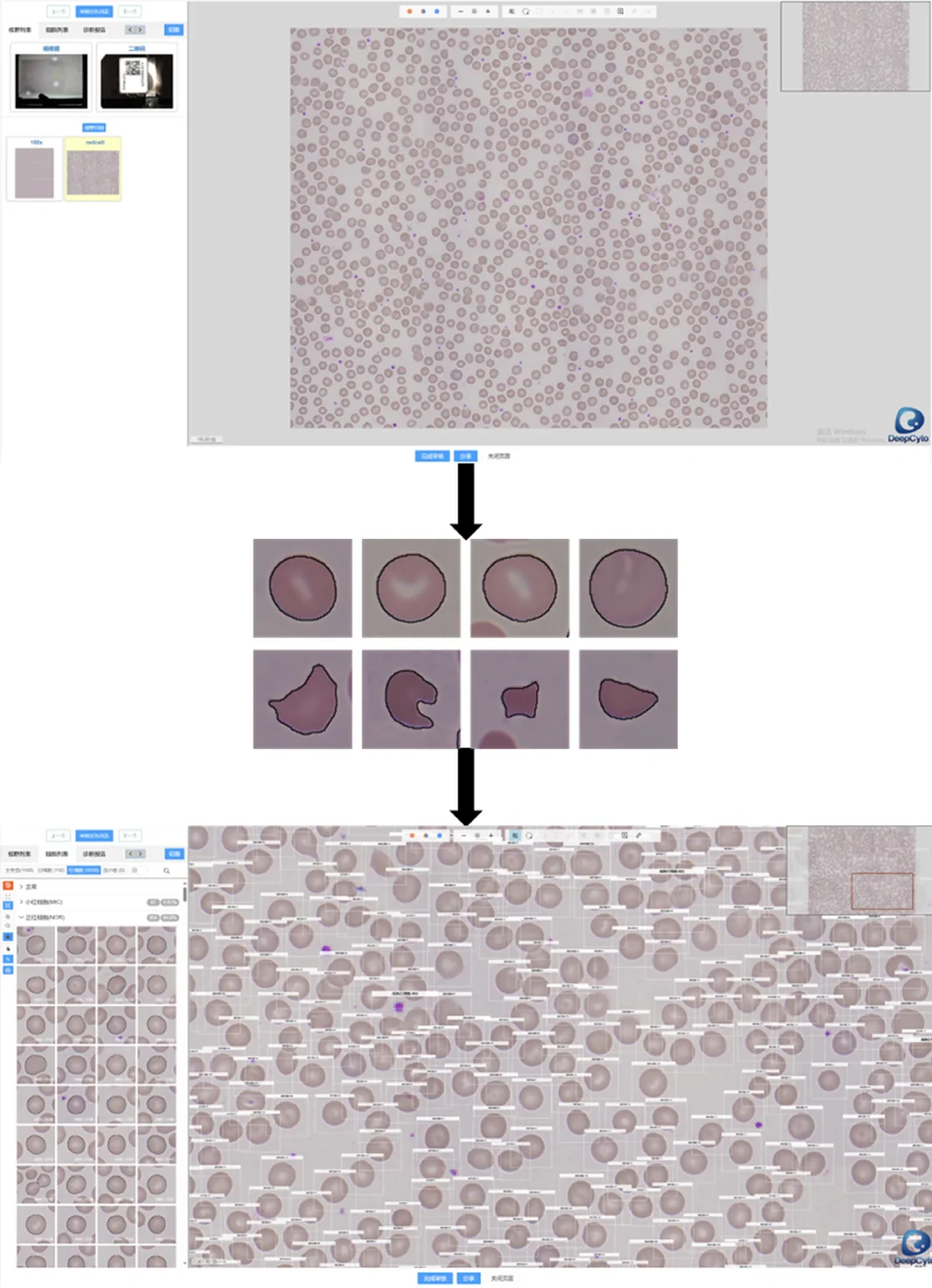
Red blood cell (RBC) contour delineation via semantic segmentation, showing precise boundary extraction of normal and fragmented RBCs.

Morphological identification of schistocytes in this study strictly complied with the original 2012 ICSH guidelines and updated 2021 ICSH standardization criteria for blood cell morphology. Two senior laboratory specialists with over 5 years of extensive experience in peripheral blood cell morphology performed independent slide review in a double‐blind manner. The identification process consisted of two sequential steps: preliminary schistocyte screening and subsequent subtype classification.

Schistocyte screening. After double‐blind evaluation, RBCs with consistent judgments from both examiners were enrolled into the schistocyte dataset, whereas discrepant cells were excluded from the cohort.

Subsequent subtype classification. True schistocytes confirmed unanimously in the primary screening underwent a second round of double‐blind subtyping following the aforementioned ICSH specifications. In cases of disagreement on subtype categorization, the two reviewers re‐evaluated cell morphology jointly and reached a consensus classification according to guideline provisions. Only subtype grouping was revised at this stage, and no previously verified true schistocytes were eliminated.

### 2.6 Computing Platform and Training Setup

All models were trained on a platform with Ubuntu 20.04 OS, 64 GB RAM, and an RTX‐3080Ti GPU. Frameworks included Python 3.11.9 and PyTorch 2.4.1.

### 2.7 Segmentation Experiment Design

Three segmentation models (U‐Net, ENet, and R2U‐Net) were evaluated (Figure S3):

- U‐Net: Classical cell segmentation framework with encoder–decoder architecture [13].
- R2U‐Net: Enhanced U‐Net with residual connections, skip connections, and recurrent blocks for boundary refinement [14].
- ENet: Lightweight model optimized for real‐time segmentation via factorized convolutions [15].

Training used a weighted loss function (pixel‐wise weighting per original U‐Net) to address cell adhesion, combined with Focal Loss to mitigate class imbalance (*α* = 0.4, *γ* = 2). The AdamW optimizer was used with an initial learning rate of 0.001 and learning rate decay over 100 epochs.

### 2.8 Classification Experiment Design

Two classification models (ResNet‐50 and XCeption) were trained under Gray and RGB modalities (Figures S4 and S5):

- ResNet‐50: Baseline model with residual connections to address gradient vanishing [16].
- XCeption: Lightweight model with depthwise separable convolutions for efficient feature extraction [17].

Images underwent contour‐based central cell extraction with background masking. Training used the AdamW optimizer (initial learning rate = 0.001, 100 epochs) and cross‐entropy loss integrated with Focal Loss (*αᵢ* set to the reciprocal of class proportion). Evaluation metrics included recall, specificity, precision, *F*_1_ score, and weighted metrics (to account for subtype imbalance).

### 2.9 Clinical Validation Design

The XCeption‐RGB model (optimal from classification experiments) was validated using the clinical dataset. Performance was evaluated using the same metrics as the classification phase, with a focus on real‐world applicability.

## 3 Results

### 3.1 Segmentation Experiment Results

R2U‐Net achieved the highest performance across key metrics: Recall = 0.868, *F*_1_ score = 0.881, mIoU = 0.807. ENet exhibited the fastest inference speed but lower accuracy, while U‐Net delivered competitive performance but lacked recurrent block‐driven boundary refinement (Tables S4 and S5).

### 3.2 Classification Experiment Results

The XCeption‐RGB model achieved the best overall performance (weighted *F*_1_ = 0.957), outperforming ResNet‐50‐Gray (0.954), ResNet‐50‐RGB (0.955), and XCeption‐Gray (0.954). RGB modality consistently outperformed grayscale across all models (Table 1; Figure 2).

**TABLE 1:** Classification performance of models under Gray and RGB modalities.

Cell type	Metric	ResNet‐50		XCeption	
		Gray	RGB	Gray	RGB
Angular cells	Recall	**0.933**	0.929	0.901	0.911
	Specificity	0.972	0.976	0.983	**0.986**
	Precision	0.910	0.920	0.940	**0.952**
	*F*_1_ score	0.921	0.924	0.920	**0.931**
Helmet cells	Recall	0.722	**0.806**	0.769	0.750
	Specificity	**0.992**	0.990	0.987	**0.992**
	Precision	0.703	0.669	0.606	**0.711**
	*F*_1_ score	0.712	**0.731**	0.678	0.730
Small fragmented RBCs	Recall	0.884	0.901	0.927	**0.936**
	Specificity	**0.988**	0.986	0.983	0.982
	Precision	**0.931**	0.921	0.913	0.907
	*F*_1_ score	0.907	0.911	0.920	**0.921**
Microspherocytes	Recall	**0.911**	0.855	0.894	0.905
	Specificity	0.996	**0.999**	0.996	0.994
	Precision	0.911	**0.962**	0.909	0.871
	*F*_1_ score	**0.911**	0.905	0.901	0.888
Normal RBCs	Recall	**0.996**	0.995	0.995	**0.996**
	Specificity	0.992	**0.993**	0.992	0.991
	Precision	**0.994**	**0.994**	**0.994**	0.993
	*F*_1_ score	**0.995**	**0.995**	0.994	**0.995**
Overall performance	Weighted *F*_1_ score	0.954	0.955	0.954	**0.957**
	Weighted recall	0.954	0.955	0.953	**0.957**
	Weighted precision	0.954	0.956	0.955	**0.958**

*Note:* The values that achieved the best performance in the table were indicated in bold.

**FIGURE 2:**
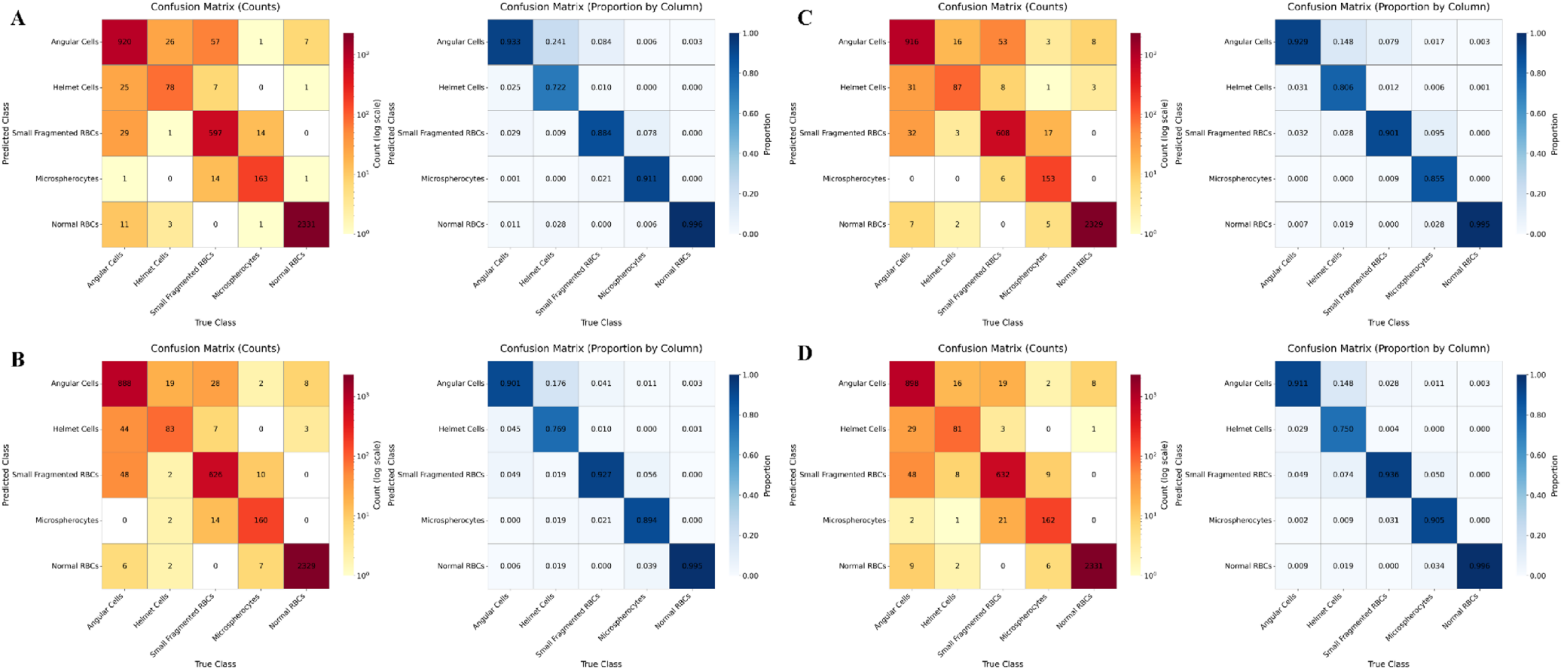
Visualization of classification results: (A) ResNet‐50 (gray); (B) XCeption (gray); (C) ResNet‐50 (RGB); (D) XCeption (RGB). Heatmaps show *F*_1_ score distribution across subtypes.

Targeted advantages of XCeption‐RGB included the following:

- Highest precision for angular cells (0.952, +3.48% vs. ResNet‐50‐RGB);
- Highest recall for small fragmented RBCs (0.936, +3.88% vs. ResNet‐50‐RGB);
- Near‐perfect recall for normal RBCs (0.996);
- Superior precision for helmet cells (0.711) compared to XCeption‐Gray (0.606), with *F*_1_ score (0.730) comparable to ResNet‐50‐RGB (0.731).

### 3.3 Clinical Validation Experimental Results

The XCeption‐RGB model achieved exceptional performance in clinical validation, with all weighted metrics reaching 0.998. Key findings included (Table 2, Figure 3):

- 100% recall for normal RBCs (*F*_1_ score = 0.999);
- 0.997 recall for small fragmented RBCs (critical for TMA diagnosis);
- 0.992 precision for helmet cells (minimizing false positives);
- 0.999 precision and 1.000 Specificity for Microspherocytes;
- 0.971 recall and 0.999 specificity for angular cells.

**TABLE 2:** Clinical validation performance of XCeption‐RGB model.

Cell type	Recall	Specificity	Precision	*F*_1_ score
Angular cells	0.971	0.999	0.926	0.948
Helmet cells	0.795	1.000	0.992	0.883
Small fragmented RBCs	0.997	0.999	0.823	0.902
Microspherocytes	0.909	1.000	0.999	0.952
Normal RBCs	1.000	0.982	0.999	0.999
Overall performance	0.998	—	0.998	0.998

*Note:* Weighted *F*_1_ score, weighted recall, and weighted precision (all 0.998) are overall performance indicators, calculated by weighting each subtype's metric by its proportion of the total cell count to account for clinical sample imbalance.

**FIGURE 3:**
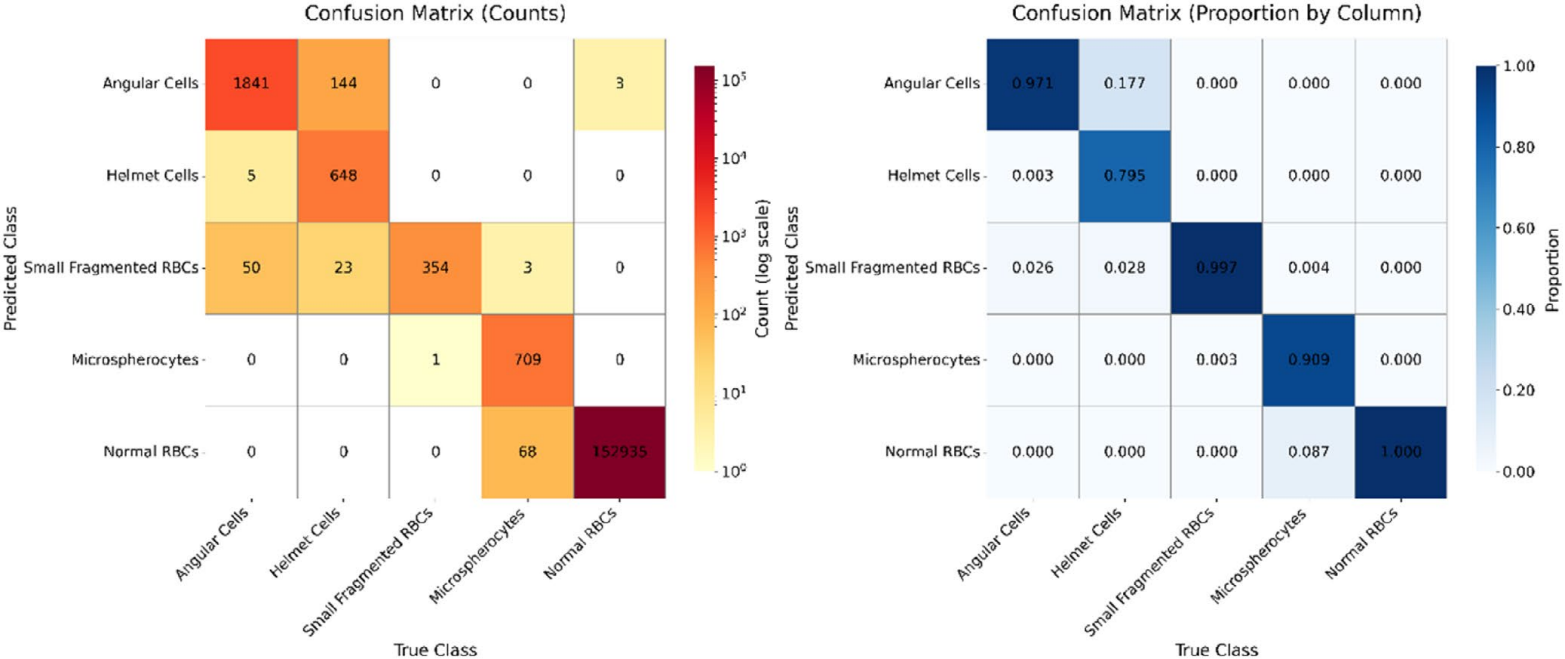
Confusion matrix (proportion by column) of clinical validation results, showing high classification accuracy across all subtypes.

## 4 Discussion

Varied counting biases arise from slide preparation and image acquisition. Uneven smearing force, angles, and blood volume deform erythrocytes to form pseudo‐schistocytes, while inconsistent thickness causes cell overlap and morphological distortion. Fluctuations in temperature, humidity, and airflow during air‐drying induce RBC shrinkage and spurious fragments. Unstable staining pH and incubation time obscure central pallor, hindering differentiation between spherocytes and microspherocytes. Improper imaging fields, defocusing, and uneven lighting also weaken the extraction of cell contour features.

Standardized protocols were adopted to mitigate these biases. All blood films were automatically prepared and stained with dynamically adjusted parameters based on hematocrit, eliminating artificial fragments caused by manual operation. Constant‐temperature directional drying and programmed staining systems stabilized experimental conditions. Images were uniformly captured in the monolayer region one‐third inward from the smear feather edge under fixed optical parameters. Nevertheless, inherent interindividual differences in blood viscosity and erythrocyte fragility cannot be fully eliminated. Even with optimal equipment settings, hyperviscous or severely anemic specimens may still produce morphological artifacts stemming from patients' underlying pathological states.

This study adopted ICSH and JSLH schistocyte classification standards, which categorize schistocytes into four subtypes: angular cells, helmet cells, small fragmented RBCs, and microspherocytes; microspherocytes are only counted as schistocytes alongside the other three types. Acanthocytes and true bite cells interfere with model training due to their similar appearance to schistocytes: acanthocytes have irregular surface spines, while bite cells feature smooth arc‐shaped indentations without sharp edges. The model distinguishes these interferents via full RBC contour extraction and built‐in morphological constraints.

Our two‐stage segmentation–classification AI pipeline outperforms end‐to‐end detectors such as YOLO [18]. Separating cell localization from subtype classification allows independent optimization of each module, a key advantage over integrated models that limit targeted tuning. Schistocyte analysis requires precise pixel‐level contour extraction and subtle subtype differentiation, which our segmentation‐first design better supports by resolving clustered, overlapping RBCs [19]. Unlike anchor‐based detectors prone to small‐object loss in NMS, R2U‐Net pixel‐level segmentation accurately isolates touching cells with an RBC recall of 0.868, supplying high‐quality data for subsequent subtype classification.

R2U‐Net integrates residual, skip, and recurrent blocks to accurately detect tiny cells and refine contours [20]. Lightweight ENet prioritizes speed over precision and fails to meet strict morphological analysis demands, while baseline U‐Net lacks recurrent modules for fragment boundary optimization. RGB‐based Xception [21] achieves superior classification via depthwise separable convolutions capturing subtle schistocyte textures and morphologies. As ICSH‐recommended diagnostic evidence, central pallor information embedded in RGB color gradients and cell structures reliably distinguishes microspherocytes, helmet cells, and normal red cells. The presence of central pallor serves as a key distinguishing trait, and multichannel RGB data greatly outperforms grayscale images in subtype identification.

Clinical validation confirmed the robust practical value of the Xception‐RGB model, with all weighted evaluation metrics reaching 0.998. This two‐stage workflow provides a standardized, reproducible alternative to manual microscopic counting by eliminating inter‐operator inconsistency and nonuniform diagnostic thresholds that limit manual detection reliability.

Hematology analyzers and AI systems differ fundamentally in detecting red cell fragments. Analyzers calculate FRC merely by cell volume and light scatter without morphological judgment [22, 23], while AI strictly follows ICSH morphological criteria to identify true schistocytes. Since FRC cannot tell genuine schistocytes apart from pseudo‐fragments like bite cells and acanthocytes, its readings are always higher than AI or manual microscopy counts. Per ICSH guidelines, FRC only rules out severe schistocytosis for TMA screening; all FRC‐positive samples require peripheral blood smear review and cannot confirm TMA alone.

## 5 Limitations and Future Directions

This study has several limitations. First, the classification dataset was derived from three TMA patients, potentially limiting generalizability to rare subtypes or TMA variants. Future work should expand the dataset to include diverse populations (e.g., HELLP syndrome, malignant hypertension). Second, helmet cell recall (0.795) was lower due to morphological similarity to other fragments and limited sample size—addressable via increased sampling and advanced augmentation (e.g., morphological warping). Third, hybrid models combining object detection efficiency with segmentation precision could reduce inference time while maintaining accuracy. Finally, integration with laboratory information systems (LIS) would enable real‐time schistocyte detection during routine CBC testing, streamlining TMA diagnostic workflows.

Our model achieves outstanding schistocyte subtyping performance on self‐built datasets, while clinical diagnosis adopts the critical cutoff of schistocyte percentage > 1%. We are prospectively enrolling clinical samples for ongoing validation, but high‐positive specimens with a schistocyte proportion above 5% are limited by single‐center sampling constraints. In the future, our hospital will collaborate with multiple medical centers to conduct multicenter research and expand the pool of high‐positive cases.

## 6 Conclusion

The two‐stage AI framework [24, 25, 26, 27] (R2U‐Net segmentation + XCeption‐RGB classification) provides a reliable, efficient solution for schistocyte identification and subtype classification. By addressing limitations of manual counting and end‐to‐end models, the system improves diagnostic accuracy, reproducibility, and efficiency—key requirements for clinical application. Its exceptional clinical performance confirms potential as a valuable auxiliary tool for TMA diagnosis, standardizing schistocyte enumeration and improving patient outcomes. Future dataset expansion and LIS integration will further enhance its utility in routine hematological practice.

## Author Contributions

D.L., Y.C., and J.Y. contributed to data collection. X.W. was responsible for figure preparation. C.F. performed algorithm analysis. L.S. drafted the manuscript. X.L. and J.L. designed the study and supervised the project.

## Funding

The study was supported by the Research Project on Undergraduate Teaching Reform of Peking University (JG2024126, 2024) and the Research Fund of Aerospace Center Hospital (YN202413, 2024).

## Ethics Statement

This study was approved by the Ethics Committee of Aerospace Center Hospital (Approval no. 2023(122), Date: 2023‐10‐08) and conducted in accordance with the Declaration of Helsinki.

## Consent

Written informed consent was obtained from the three patients, and written authorization was also secured for the use of their relevant clinical data and laboratory test report data.

## Conflicts of Interest

The authors declare no conflicts of interest.

## Supporting information


**Table S1:** Sample statistics for RBC segmentation dataset.
**Table S2:** Sample statistics for schistocyte classification dataset.
**Table S3:** Sample statistics for clinical validation dataset.
**Table S4:** Inference time of segmentation models (640 × 512 pixel image).
**Table S5:** Segmentation performance metrics.

**Figure S1:** Classification of schistocyte subtypes based on ICSH guidelines.

**Figure S2:** Peripheral blood smear scanned image (100× magnification) showing uniform cell distribution and clear morphological details.

**Figure S3:** Architectural diagrams of segmentation models: (A) U‐Net; (B) R2U‐Net; (C) ENet.

**Figure S4:** Architectural diagrams of classification models: (A) ResNet‐50; (B) XCeption.

**Figure S5:** Representative 2 × 2 arrays of schistocyte subtype images under RGB (left) and grayscale (right) modalities, showing color/texture differences.

## Data Availability

The data that support the findings of this study are available from the corresponding author upon reasonable request.
