# Supplementary material for: AI‐Enabled Automated Schistocyte Classification in Peripheral Blood for TMA Auxiliary Diagnosis
Source: Int J Lab Hematol. 2026 Aug 5;48(6):1486–93. doi: 10.1111/ijlh.70209 (PMC13729982; doi:10.1111/ijlh.70209)
Supplement: Supplementary file 1 [file IJLH-48-1486-s001.docx]

Table S1. Sample Statistics for RBC Segmentation Dataset

| Number of Patients | Number of Smears | Training Set RBCs | Test Set RBCs | Total RBCs |
| --- | --- | --- | --- | --- |
| 183 | 183 | 19368 | 5699 | 25067 |

Abbreviations: RBCs = red blood cells.

Table S2. Sample Statistics for Schistocyte Classification Dataset

| Group | Angular Cells | Helmet Cells | Small Fragmented RBCs | Microspherocytes | Total Schistocytes | Normal RBCs | Total Cells |
| --- | --- | --- | --- | --- | --- | --- | --- |
| Training Set | 5,392 | 843 | 3,930 | 993 | 11,158 | 13,120 | 24,278 |
| Test Set | 990 | 112 | 682 | 183 | 1,967 | 2,341 | 4,308 |
| Total | 6,382 | 955 | 4,612 | 1,176 | 13,125 | 15,461 | 28,586 |

Abbreviations: RBCs = red blood cells.

Table S3. Sample Statistics for Clinical Validation Dataset

| Cell Type | Angular Cells | Helmet Cells | Small Fragmented RBCs | Microspherocytes | Total Schistocytes | Normal RBCs | Total Cells |
| --- | --- | --- | --- | --- | --- | --- | --- |
| Clinical Validation | 1,896 | 815 | 355 | 780 | 3,846 | 152,938 | 156,784 |

Abbreviations: RBCs = red blood cells.

Table S4. Inference Time of Segmentation Models (640×512 Pixel Image)

| Metric | U-Net | ENet | R2U-Net |
| --- | --- | --- | --- |
| Inference Time (ms) | 12.2 | 17.5 | 14.1 |

Note: Inference time excludes preprocessing steps, measured on the same computing platform.

Table S5. Segmentation Performance Metrics

| Model | Recall | Precision | F1-Score | mIoU |
| --- | --- | --- | --- | --- |
| U-Net | 0.857 | **0.898** | 0.877 | 0.801 |
| ENet | 0.770 | 0.890 | 0.826 | 0.732 |
| R2U-Net | **0.868** | 0.895 | **0.881** | **0.807** |
